# Effects of sire line, birth weight and sex on growth performance and carcass traits of crossbred pigs under standardized environmental conditions

**DOI:** 10.5194/aab-63-367-2020

**Published:** 2020-11-03

**Authors:** Kathrin Elbert, Neal Matthews, Ralf Wassmuth, Jens Tetens

**Affiliations:** 1 Department of Animal Sciences, Division of Functional Breeding, Georg-August University Göttingen, Burckhardtweg 2, 37077 Göttingen, Germany; 2 Pig Improvement Company (PIC) North America, 100 Bluegrass Commons Blvd., Ste. 2200, Hendersonville, TN 37075, USA; 3 Faculty of Agricultural Sciences and Landscape Architecture, Division Animal Breeding, University of Applied Sciences Osnabrück, Am Kruempel 31, 49090 Osnabrück, Germany

## Abstract

A variety of available terminal sire lines makes the choice of terminal sire line complex for the pig producer. Higher birth weights are important for subsequent growth performance and selection for this trait is also necessary in sire lines. The aim was to investigate the effect of sire line, birth weight and gender on growth performance, carcass traits and meat quality. In total 3844 crossbred pigs from Camborough Pig Improvement Company (PIC) dams matched with either a Synthetic (A) or Piétrain (B) sire line were used. Pigs from line A grew faster (p<0.01), showed higher feed intake (p<0.01) and reached a higher final body weight (p≤0.01), but they had a similar efficiency (p=0.179). Leaner carcasses and heavier primal cuts (p<0.001) were observed in pigs from line B. Carcasses from pigs sired by line A had higher meat quality (p<0.001). Males had a higher growth rate (p≤0.05) but had a poorer feed efficiency (p<0.01). Heavier birth weight pigs and females had leaner, higher value carcasses with heavier primal cuts (p<0.001) compared to middle and low birth weight females or males. Sire line by sex interactions was significant for growth (p≤0.05) and carcass traits (p<0.001). Interaction between sire line and birth weight classes were only detected for loin depth (p<0.01). Line A is preferable if the numbers of fatting pigs per fattening place and year should be improved, and line B is an option to increase leanness and carcass primal cuts.

## 1 Introduction

Production efficiency of fattening pigs in terms of average daily gain, feed efficiency and carcass composition is a heritable trait complex (Aymerich et al., 2019; Cámara et al., 2016; Latorre et al., 2003; Serrano et al., 2008), for which considerable heterosis effects are harnessed in crossbreeding schemes (García-Casco et al., 2012; Katoele et al., 2002; Luo et al., 2018). In commercial pig production, the dam line is usually fixed, and as a consequence of artificial insemination (AI) sire line selection is flexible. According to the BMEL (2019), currently nine pig breeding companies are operating in the European Union. The fact that each company improves and offers at least one or two basic sire lines, and selects these in different ways, leads to a variety of sire lines. This results in a complex choice for the pig producers.

Individual piglet birth weight is important for subsequent growth performance (Fix et al., 2010; Vázquez-Gómez et al., 2020). Sire line impacted piglet birth weight (Vermeulen et al., 2016); hence, a selection of purebred sires on birth weight is useful (Dufrasne et al., 2013), and the importance of sire lines in breeding programs is increasing (Dodenhoff et al., 2019). Regarding gender, controversial results with respect to growth performance can be found (Franco et al., 2014; Lee et al., 2019; Morales et al., 2011). Hence, a closer examination of sire line effects on offspring birth weight and of the implications of gender for further performance is needed.

Several studies have analyzed offspring performance in the period of fattening with various sire lines, in terms of variables such as feed composition, different breeding values and selection strategies, or various possibilities of male castration (Cámara et al., 2016; Cilla et al., 2006; De Cuyper et al., 2019; Gilleland et al., 2019; Lowell et al., 2019; Morales et al., 2011). Against this background, an integrated view on productivity from birth to finishing seems necessary. Hence, in the current study, different sire lines where mated to the same sow genotype in an integrated production system. The aim was to investigate the effect of sire line, birth weight, and gender on growth performance, carcass and meat quality traits.

## 2 Materials and methods

### 2.1 Ethics statement

All work was done in accordance with the German legal and ethical requirements for appropriate animal procedures. This study was approved by the institutional animal care and use committee of Göttingen University under file number E8-19.2.1 Husbandry and diets

A total of 3844 crossbred pigs divided into four batches resulted from the mating of 337 dams (Camborough Pig Improvement Company, PIC, a crossbred sow between Large White and Landrace, F1) to 35 terminal sires (sire line A: 15 boars; sire line B: 20 boars). Line A was the PIC337 (PIC, 100 Bluegrass Commons Blvd, Ste 2200, Hendersonville, TN 37075), which is a closed composite population for over 40 years that includes genes of Duroc, Landrace, Large White and Piétrain descent and has been selected for lean efficient growth and carcass value. A pure Piétrain population was Line B, the PIC408 (PIC, 100 Bluegrass Commons Blvd, Ste 2200, Hendersonville, TN 37075), which is selected for carcass value, growth and efficiency. The batches were organized over four consecutive fattening cycles between May 2018 and February 2019. All pigs were born, reared and fattened under the same standardized environmental conditions in a commercial closed herd system. The farm was approximately 50 km west of Leipzig, Germany, and consisted of 3100 fattening places. At birth, all pigs received an individual transponder ear tag. On their third day, males were given an intramuscular injection of meloxicam (Metacam^®^, 5 mg mL${}^{-1}$) and were surgically castrated. At 71.9±1.1 days of age, pigs were regrouped into the common finishing barn. All pigs were placed in groups of 40 to 64 pigs per pen according to the genetic sire line and gender. Pens were stocked to the same number of pigs per square meter and pig size. Pigs were selected for slaughtering by an estimation of the individual body weight. The weight estimation was determined by a pen reference group, consisting of two pigs, which had reached the final body condition. These pigs were weighed individually with an animal scale. Based on this reference group the other pigs were selected and their weights were estimated. Pen-wise and at each selection day for slaughtering this process step was repeated by the same person. Pens had a slatted floor and cross feeder. Space allowance was 1 m${}^{2}$ per pig. Pigs had ad libitum access to water. They were fed with a sensor feeding system that supplied diets appropriate to the fattening period. Table 1 presents the compositions of the diets. The average ambient temperature during the fattening period was 22.7±5.1 ${}^{\circ }$C. Temperature was controlled and adjusted according to the outside environment temperature by means of an automatic indoor heating and ventilation system.

**Table 1:** Composition and nutrient content of the pig diets (%, as-fed basis, unless otherwise indicated).

	Fattening phase (d)			
	0 to 10	11 to 30	31 to 69	70 to harvest
Ingredient${}^{\text{a}}$				
Wheat	21.39	10.71	8.03	4.58
Barley	16.70	10.50	8.56	7.76
Corn-Cob mix	23.61	31.04	32.51	34.65
Molasses chip	2.77	3.18	3.17	3.17
Soybean meal	14.60	11.93	10.58	7.68
Elan 1480	5.86	5.09	4.89	4.88
Wheat starch	7.81	13.04	15.64	18.22
Wheat slop	3.04	6.09	6.08	7.29
Rye slop	4.21	8.43	10.54	11.78
Nutrient content${}^{\text{b}}$				
ME, Pig (MJ kg${}^{-1}$)	13.02	13.08	13.07	13.05
Crude protein	17.17	16.86	16.47	15.67
Crude fiber	3.28	3.26	3.22	3.18
Crude fat	2.63	3.01	3.07	3.20
Crude ash	5.99	5.64	5.52	5.45
Lysine	1.13	1.06	1.02	0.97
Methionine, Cysteine	0.67	0.65	0.63	0.61
Tryptophan	0.21	0.20	0.19	0.18
Threonine	0.66	0.65	0.63	0.60
Calcium	0.83	0.74	0.72	0.72
Phosphorous	0.56	0.57	0.57	0.58
Sodium	0.25	0.24	0.25	0.26
Vitamin A (IE)	6302	5470	5258	5248
Vitamin D (IE)	1547	1343	1291	1288
Vitamin E (mg)	97	85	81	81
Phytase (FYT)	688	597	574	572
Lysine 10${}^{-1}$ MJ ME, pig	0.87	0.81	0.78	0.74
Lysine: Met + Cys	1:0.59	1:0.61	1:0.62	1:0.63
Lysine: Tryptophan	1:0.19	1:0.19	1:0.19	1:0.18
Lysine: Threonine	1:0.59	1:0.61	1:0.62	1:0.63

${}^{\text{a}}$ Represented as % of 100 % dry matter. ${}^{\text{b}}$ Represented as % of 88 % dry matter.

### 2.2 Growth performance

Individual birth weights (BW${}_{\text{birth}}$) were recorded within 12–24 h after birth. After regrouping in the fattening barn, all pigs were weighed by pen, and an average initial body weight per pen was calculated (BW${}_{\text{initial}}$). This date was assigned as day 0, the first day of the fattening phase on which feed consumption per pen was recorded. A single day before slaughtering the selected pigs were weighed by pen to calculate the average final body weight (BW${}_{\mathrm{final}}$). These data were used to calculate average daily gain (ADG), average daily feed intake (ADFI) and the average feed conversion ratio (FCR) for each pen. The age at slaughter and the hot carcass weight (HCW) were used to calculate the individual net ADG (ADG${}_{\text{net}}$) per pig.

### 2.3 Carcass traits

Pigs were transported approximately 35 km to the commercial slaughterhouse Tönnies Zerlegungs GmbH, Weissenfels, Germany. Access to feed was stopped 12 h before slaughter although water was still provided ad libitum. Offspring of both sire lines were slaughtered on the same day. Upon arrival at the slaughterhouse, pigs were rested for 3 h, with access to water. All pigs were slaughtered in accordance with Germany's current regulations on animal welfare and protection for the slaughter process (TierSchIV, 2012). Carcass composition (n=3437 pigs) was determined using Auto-FOM III and composition traits variables included HCW, lean meat (LM), loin depth (LD), back fat thickness (FT), belly weight (BE), belly lean meat percentage (BLM), boneless ham weight (BH), loin weight (LO), boneless loin weight (BLO) and boneless shoulder weight (BSH).

### 2.4 Meat quality

Investigation of the meat quality was conducted due to pH measurements, whereby both genotypes were measured at the same slaughter days. The pH was measured at the M. longissimus dorsi at 45 min (pH${}_{\text{45 min}}$) and 24 h (pH${}_{\text{24 h}}$) post mortem, on the left side of the carcass between the 10th and 11th rib. The electrode of the pH/ORP meter (HI 98160 pH/ORP meter, HANNA instruments) was calibrated with buffers at pH of 4 and 7 immediately prior to taking measurements. Based on carcass temperature, the pH meter was standardized to 40.0 ${}^{\circ }$C for pH${}_{\text{45 min}}$ and to 4.0 ${}^{\circ }$C for pH${}_{\text{24 h}}$. Post mortem, after dressing and recording pH${}_{\text{45 min}}$, carcasses were suspended in air and chilled to a temperature of 4.0 ${}^{\circ }$C.

### 2.5 Statistical analyses

Statistical analyses were performed using SAS version 9.4 (SAS Institute Inc., Cary NC, USA). A descriptive analysis (mean, standard deviation, and minimum and maximum values) was carried out with the MEANS procedure. Multivariate analysis of variances were performed by the procedure MIXED with the REML method and using the Kenward–Roger adjustment for the degrees of freedom. The Tukey test was used to compare the least-squares means (LSQ means) with the standard error (SE) for each trait. Differences were considered statistically significant at a confidence level of 95 % (p≤0.05). Appropriate two-way interactions between the fixed effects were tested and dropped from the model at a p value of >0.05 in the F test. For growth performance traits (ADG, ADFI, FCR, BW${}_{\text{initial}}$, BW${}_{\mathrm{final}}$) pen was considered as the experimental unit, except for ADG${}_{\text{net}}$ and BW${}_{\text{birth}}$. These, as well as carcass traits (HCW, LM, LD, FT, BE, BLM, BH, LO, BLO, BSH) and meat quality traits (pH${}_{\text{45 min}}$ and pH${}_{\text{24 h}}$), were analyzed based on individual measurements per pig. According to the quantiles of the frequency distribution, BW${}_{\text{birth}}$ (1.45±0.35 kg, min: 0.38 kg; max: 2.65 kg) was divided into three groups (BW${}_{\text{Q}}$): lower 25 % (<1.22 kg; BW${}_{\text{Q1}}$), middle 50 % (≥1.22<1.70 kg, $\tilde{x}=1.46$ kg; BW${}_{\text{Q2}}$) and upper 25 % (≥1.70 kg; BW${}_{\text{Q3}}$). BW${}_{\text{Q}}$ was used as fixed effect for carcass traits and ADG${}_{\text{net}}$. Moreover, sire line (G = Line A, Line B), gender (S = male, female) and batch (B = 1, 2, 3, 4) were used as fixed effects for all observed traits. Pen was used as random effect within sire line and batch (Pen${}_{\left(G\times B\right)}$) for growth traits. In commercial swine production, typically a defined time allotment for various phases of production is applied and initial age (AI), slaughter age (ASL) and HCW differed significant between the sire lines. Hence, it was adjusted to 72 d (AI${}_{\text{adj}}$), to 173 d (ASL${}_{\text{adj}}$) and to 91 kg (HCW${}_{\text{adj}}$), respectively. In order to correct for age or HCW, these were included as linear covariables. BW${}_{\mathrm{final}}$ was introduced into the models for ADG, ADFI and FCR as a linear covariable. Table 2 contains all used effects that were included in the final models for growth and carcass traits.

**Table 2:** Final models with the p values of fixed effects, random effects, covariates and interactions for growth traits, carcass traits and meat quality.

	Fixed				Random	Covariates				Interaction		
	G	S	B	BW${}_{\text{Q}}$	Pen${}_{\left(G\times B\right)}$	BW${}_{\mathrm{final}}$	AI${}_{\text{adj}}$	ASL${}_{\text{adj}}$	HCW${}_{\text{adj}}$	G×S	G×B	$G\times {\mathrm{BW}}_{\text{Q}}$
Growth trait												
BW${}_{\text{initial}}$	0.743	0.495	0.245	0.366	0.166							
BW${}_{\mathrm{final}}$	${}^{**}$	${}^{***}$	${}^{***}$	${}^{***}$	0.197							
ADG${}_{\text{net}}$	${}^{***}$	${}^{***}$	${}^{***}$	${}^{***}$	${}^{***}$	${}^{**}$						
ADG	${}^{**}$	${}^{*}$	${}^{*}$	${}^{***}$	${}^{**}$	${}^{*}$						
ADFI	${}^{**}$	${}^{***}$	${}^{***}$	0.975	${}^{***}$	${}^{**}$	${}^{*}$					
FCR	0.179	${}^{**}$	${}^{***}$	${}^{***}$	0.840							
Age${}_{\text{initial}}$	${}^{***}$	0.268	${}^{***}$							${}^{***}$		
Age${}_{\text{SL}}$	${}^{***}$	${}^{***}$	${}^{***}$							${}^{**}$	${}^{***}$	
Carcass trait												
HCW	${}^{***}$	${}^{***}$	${}^{***}$	${}^{***}$				${}^{***}$		${}^{***}$	${}^{***}$	
LM	${}^{***}$	${}^{***}$	${}^{***}$	${}^{***}$					${}^{***}$	${}^{***}$	${}^{***}$	
LD	${}^{***}$	${}^{***}$	${}^{***}$	${}^{***}$					${}^{***}$	${}^{***}$	${}^{***}$	${}^{**}$
FT	0.423	${}^{***}$	${}^{***}$	${}^{***}$					${}^{***}$	${}^{***}$	${}^{***}$	
BE	${}^{***}$	${}^{***}$	${}^{***}$	${}^{***}$					${}^{***}$	${}^{***}$	${}^{***}$	
BLM	${}^{***}$	${}^{***}$	${}^{***}$	${}^{***}$					${}^{***}$	${}^{***}$	${}^{***}$	
BH	${}^{***}$	${}^{***}$	${}^{***}$	${}^{***}$					${}^{***}$	${}^{***}$	${}^{***}$	
LO	${}^{***}$	${}^{***}$	${}^{***}$	${}^{***}$					${}^{***}$	${}^{***}$	${}^{***}$	
BLO	${}^{***}$	${}^{***}$	${}^{***}$	${}^{***}$					${}^{***}$	${}^{***}$	${}^{***}$	
BSH	${}^{***}$	${}^{***}$	${}^{***}$	${}^{***}$					${}^{***}$	${}^{***}$	${}^{***}$	
Meat quality												
pH${}_{\text{45 min}}$	0.061	0.239	${}^{***}$						0.862			
pH${}_{\text{24 h}}$	${}^{***}$	0.444	${}^{***}$						${}^{***}$		${}^{**}$	

${}^{*}$ p≤0.05. ${}^{**}$ p<0.01. ${}^{***}$ p<0.001.

## 3 Results

The descriptive statistics of all investigated traits according the sire line are in Table 3. A total of 73 pens were used to calculate growth performance. ADG${}_{\text{net}}$ and carcass traits were based on 3437 carcasses. Overall, 672 pH${}_{\text{45 min}}$ and 1895 pH${}_{\text{24 h}}$ measurements were collected. The LSQ means according to the sire line are reported in Table 4. Offspring of both sire lines exhibited the same BW${}_{\text{initial}}$ (p=0.743). During the fattening phase, pigs sired by line A grew 48.8 g d${}^{-1}$ faster (p<0.01) and reached 16.5 g d${}^{-1}$ higher ADG${}_{\text{net}}$ (p<0.001) as compared to pigs sired by line B. A 60 g d${}^{-1}$ higher ADFI (p<0.01), but the same feed efficiency as sire line B (p=0.179) was detected at sire line A. These offspring were 3.3 kg heavier in BW${}_{\mathrm{final}}$ (p<0.01). Based on a 90.0 kg weight gain at finishing, 6 fewer fatting days were needed. Regarding the carcass traits, HCW was 1.3 kg higher for offspring of sire line A based on the same age at slaughtering. Sire line A had 340 g more BE (p<0.001) but no difference in FT (p=0.423) compared to pigs from sire line B. Pigs sired by line B had a leaner carcass with heavier primal cuts (p<0.001). No difference between sire lines (p=0.061) was detected for pH${}_{\text{45 min}}$, but offspring sired by line B had a lower pH${}_{\text{24 h}}$ (p<0.001).

**Table 3:** Descriptive statistics with number of observations, mean and standard deviation for growth traits, carcass traits, and meat quality according to the sire line.

	Sire line A			Sire line B		
	n	Mean	SD	n	Mean	SD
Growth traits						
ADG${}_{\text{net}}$ (g d${}^{-1}$)	1597	534.10	56.55	1840	517.67	52.87
ADG (g d${}^{-1}$)	33	899.45	51.44	40	835.18	88.28
ADFI (kg d${}^{-1}$)	33	2.29	0.21	40	2.20	0.24
FCR (kg kg${}^{-1}$)	33	2.53	0.18	40	2.60	0.20
BW${}_{\text{initial}}$ (kg)	33	27.17	2.82	40	27.80	2.38
BW${}_{\text{final}}$ (kg)	33	115.93	5.11	40	112.29	6.04
age${}_{\text{initial}}$ (d)	1597	71.69	1.03	1840	72.14	1.12
age${}_{\text{SL}}$ (d)	1597	171.65	5.87	1840	174.30	5.76
Carcass traits						
HCW (kg)	1597	91.50	8.51	1840	90.07	8.11
LM (%)	1597	59.74	3.26	1840	60.45	3.37
LD (mm)	1597	62.68	4.35	1840	66.50	4.88
FT (mm)	1597	14.03	2.71	1840	13.83	2.66
BE (kg)	1597	13.27	1.67	1840	12.67	1.62
BLM (%)	1597	57.27	4.20	1840	58.16	4.38
BH (kg)	1597	17.47	1.63	1840	17.82	1.60
LO (kg)	1597	10.98	1.06	1840	11.04	1.02
BLO (kg)	1597	6.86	0.75	1840	7.05	0.74
BSH (kg)	1597	8.65	0.74	1840	8.59	0.71
Meat quality						
pH${}_{\text{45 min}}$	271	6.51	0.27	401	6.55	0.23
pH${}_{\text{24 h}}$	872	5.43	0.14	1023	5.39	0.14

**Table 4:** LSQ mean (with SE as index) for growth traits, carcass traits and meat quality according to the sire line.

	Sire line	
	A	B
Growth traits		
BW${}_{\text{initial}}^{1}$ (kg)	27.61${}_{0.53}$	27.36${}_{0.41}$
BW${}_{\mathrm{final}}^{2}$ (kg)	115.34${}_{0.84}^{\text{a}}$	112.03${}_{0.73}^{\text{b}}$
ADG${}_{\text{net}}$ (g d${}^{-1}$)	531.11${}_{3.09}^{\text{a}}$	514.65${}_{2.93}^{\text{b}}$
ADG (g d${}^{-1}$)	890.01${}_{10.51}^{\text{a}}$	841.20${}_{9.87}^{\text{b}}$
ADFI (kg d${}^{-1}$)	2.27${}_{0.01}^{\text{a}}$	2.21${}_{0.01}^{\text{b}}$
FCR (kg kg${}^{-1}$)	2.54${}_{0.02}$	2.59${}_{0.02}$
Carcass traits		
HCW${}^{2}$ (kg)	91.08${}_{0.18}^{\text{a}}$	89.83${}_{0.17}^{\text{b}}$
LM${}^{3}$ (%)	59.75${}_{0.07}^{\text{a}}$	60.40${}_{0.06}^{\text{b}}$
LD${}^{3}$ (mm)	62.51${}_{0.09}^{\text{a}}$	66.87${}_{0.09}^{\text{b}}$
FT${}^{3}$ (mm)	13.99${}_{0.05}$	13.93${}_{0.05}$
BE${}^{3}$ (kg)	13.18${}_{0.02}^{\text{a}}$	12.84${}_{0.01}^{\text{b}}$
BLM${}^{3}$ (%)	57.27${}_{0.09}^{\text{a}}$	58.10${}_{0.08}^{\text{b}}$
BH${}^{3}$ (kg)	17.83${}_{0.02}^{\text{a}}$	17.97${}_{0.02}^{\text{b}}$
LO${}^{3}$ (kg)	10.92${}_{0.01}^{\text{a}}$	11.16${}_{0.01}^{\text{b}}$
BLO${}^{3}$ (kg)	6.82${}_{0.01}^{\text{a}}$	7.12${}_{0.01}^{\text{b}}$
BSH${}^{3}$ (kg)	8.60${}_{0.01}^{\text{a}}$	8.68${}_{0.01}^{\text{b}}$
Meat quality		
pH${}_{\text{45 min}}^{3}$	6.51${}_{0.02}$	6.54${}_{0.02}$
pH${}_{\text{24 h}}^{3}$	5.47${}_{0.01}^{\text{a}}$	5.43${}_{0.01}^{\text{b}}$

${}^{1}$ Initial age adjusted to 72 d. ${}^{2}$ Slaughter age adjusted to 173 d. ${}^{3}$ HCW adjusted to 91 kg. ${}^{\text{a–b}}$ Values within a row with different superscripts differ significantly.

Males grew faster (ADG: 883.37 vs. 847.83 g d${}^{-1}$, p≤0.05; ADG${}_{\text{net}}$: 532.64 vs. 513.11 g d${}^{-1}$, p<0.001) and had a higher feed intake (ADFI: 2.31 vs. 2.16 kg d${}^{-1}$, p<0.001) but were less efficient (FCR: 2.62 vs. 2.51 kg kg${}^{-1}$, p<0.01) compared to females. Consequently, males reached a higher BW${}_{\mathrm{final}}$ (115.56 vs. 111.80, p<0.001) and a 2.05 kg heavier HCW (p<0.001). Females had a better carcass value and were 3.04 % leaner with 2.39 mm thicker LD and 396 g less BE (p<0.001) but had heavier primal cuts (p<0.001). Neither pH${}_{\text{45 min}}$ nor pH${}_{\text{24 h}}$ were affected by sex (p≥0.05). The LSQ means of the significant interactions between sire line and sex are presented in Table 5. Males and females sired by line A did not differ in ADG${}_{\text{net}}$ and ADG (p≥0.05). In sire line B a faster growth (p<0.01) and higher feed intake (p<0.01) were detected for males compared to females. HCW of males sired by this line did not differ from males and females of sire line A. Females of sire line B were lighter in HCW compared to all other sex and genotype variations. Regardless of sire line, similar ADG${}_{\text{net}}$, ADG, ADFI, HCW, LM, FT, BLM and BSH between males was observed (p≥0.05). Females sired from line B reached heavier BH, LO, BLO and BSH compared to the other sex and genotype variations.

**Table 5:** LSQ mean (with SE as index) of the significant interactions between sire line and sex for growth traits and carcass traits.

	Sire line A		Sire line B	
	Male	Female	Male	Female
Growth traits				
ADG${}_{\text{net}}$ (g d${}^{-1}$)	536.00${}_{3.90}^{\text{a}}$	526.72${}_{3.97}^{\text{a}}$	528.69${}_{3.79}^{\text{a}}$	499.49${}_{3.88}^{\text{b}}$
ADG (g d${}^{-1}$)	893.21${}_{14.9}^{\text{a}}$	886.79${}_{14.4}^{\text{a}}$	873.54${}_{13.0}^{\text{a}}$	808.87${}_{15.3}^{\text{b}}$
ADFI (kg d${}^{-1}$)	2.31${}_{0.02}^{\text{a}}$	2.21${}_{0.02}^{\text{b}}$	2.31${}_{0.02}^{\text{a}}$	2.11${}_{0.02}^{\text{c}}$
Carcass traits				
HCW${}^{1}$ (kg)	91.66${}_{0.26}^{\text{a}}$	90.52${}_{0.26}^{\text{b}}$	91.31${}_{0.24}^{\text{ab}}$	88.35${}_{0.25}^{\text{c}}$
LM${}^{2}$ (%)	58.49${}_{0.09}^{\text{a}}$	61.01${}_{0.09}^{\text{b}}$	58.64${}_{0.09}^{\text{a}}$	62.17${}_{0.09}^{\text{c}}$
LD${}^{2}$ (mm)	61.50${}_{0.13}^{\text{a}}$	63.48${}_{0.13}^{\text{b}}$	65.40${}_{0.13}^{\text{c}}$	68.31${}_{0.13}^{\text{d}}$
FT${}^{2}$ (mm)	14.81${}_{0.07}^{\text{a}}$	13.07${}_{0.07}^{\text{b}}$	15.17${}_{0.07}^{\text{a}}$	12.70${}_{0.07}^{\text{c}}$
BE${}^{2}$ (kg)	13.34${}_{0.02}^{\text{a}}$	13.02${}_{0.02}^{\text{b}}$	13.07${}_{0.02}^{\text{bc}}$	12.60${}_{0.02}^{\text{d}}$
BLM${}^{2}$ (%)	55.67${}_{0.12}^{\text{a}}$	58.88${}_{0.12}^{\text{b}}$	55.78${}_{0.11}^{\text{a}}$	60.41${}_{0.12}^{\text{c}}$
BH${}^{2}$ (kg)	17.05${}_{0.03}^{\text{a}}$	17.70${}_{0.03}^{\text{b}}$	17.52${}_{0.03}^{\text{c}}$	18.43${}_{0.03}^{\text{d}}$
LO${}^{2}$ (kg)	10.75${}_{0.01}^{\text{a}}$	11.08${}_{0.01}^{\text{b}}$	10.92${}_{0.01}^{\text{c}}$	11.40${}_{0.01}^{\text{d}}$
BLO${}^{2}$ (kg)	6.66${}_{0.01}^{\text{a}}$	6.99${}_{0.01}^{\text{b}}$	6.90${}_{0.01}^{\text{c}}$	7.35${}_{0.01}^{\text{d}}$
BSH${}^{2}$ (kg)	8.50${}_{0.01}^{\text{a}}$	8.71${}_{0.01}^{\text{b}}$	8.51${}_{0.01}^{\text{a}}$	8.83${}_{0.01}^{\text{c}}$

${}^{1}$ Slaughter age adjusted to 173 d. ${}^{2}$ HCW adjusted to 91 kg. ${}^{\text{a–d}}$ Values within a row with different superscripts differ significantly (p<0.01).

Growth rate and carcass traits according to the birth weight classes are presented in Table 6. Low birth weight pigs (BW${}_{\text{Q1}}$) had the lowest ADG${}_{\text{net}}$ compared to pigs of the middle (BW${}_{\text{Q2}}$) and the heaviest (BW${}_{\text{Q3}}$) birth weight classes. Pigs in the BW${}_{\text{Q3}}$ reached the fastest growth rate (p<0.001). Moreover, pigs HCW differed correspondingly among the birth weight groups (p<0.001). Pigs in BW${}_{\text{Q1}}$ were 4.66 kg lighter compared to pigs of BW${}_{\text{Q2}}$ and 5.82 kg lighter than pigs belonging to BW${}_{\text{Q3}}$. Fatter carcasses based on a thicker FT and a heavier BE, as well as a lower percentage in LM and BLM, were found for pigs of BW${}_{\text{Q1}}$ but not for pigs of BW${}_{\text{Q2}}$ and BW${}_{\text{Q3}}$ (p<0.001). Furthermore, birth weight was positively related to the weight of BH, LO, BLO and BSH, and a difference was detected among the BW${}_{\text{Q}}$ (p<0.001). The interaction between BW${}_{Q}$ × sire line was significant for LD (p<0.01), in sire line A, similar LD was detected for pigs of BW${}_{\text{Q2}}$ and BW${}_{\text{Q3}}$ (62.79 vs. 62.98 mm, p≥0.05), whereby all other BW${}_{\text{Q}}$ and genotype variations differed (p<0.001).

## 4 Discussion

Higher ADG was observed for pigs sired by sire line A, which is a reason for the heavier BW${}_{\mathrm{final}}$ at a fixed slaughter age and may enable more pigs per fattening place and year. This is in line with several other studies applying the same approach (Aymerich et al., 2019; Cámara et al., 2016; Lee et al., 2019; Serrano et al., 2008), in which differences between 2 and 6 kg for BW${}_{\mathrm{final}}$ (p≤0.05) and 41 and 152 g d${}^{-1}$ for ADG (p≤0.05) among crossbred pigs were observed. It is to be expected that the higher ADFI for pigs sired by line A is linked to the faster growth rate of these offspring. This assumption is generally in line with Kavlak and Uimari (2019) and Labroue et al. (1997), who reported a positive correlation between feed consumption per visit and growth rate. A greater emphasis on growth in line A in competition with line B is presumed. As a consequence of faster growth, a heavier belly weight and a fatter carcass of crossbreeds sired by line A were detected. According to Kavlak and Uimari (2019), a positive genetic correlation between ADFI and back fat thickness (r=0.60–0.72) does exist. Consequently, pigs consuming more feed tended to gain more fat. A limited protein disposition capacity of pigs sired by line A with respect to the high feed intake could provide an explanation for the fat accumulation in the carcass and is generally in agreement with finding of Hermesch et al. (2000). Additionally, a higher ADFI is linked with a poorer feed efficiency (Godinho et al., 2018a). Feed efficiency was equal for both lines (p=0.179), which indicates comparable energy demands for maintenance. But in consideration of the difference in carcass composition it is to expect that the protein requirements differ between the sire lines. In particular, it is well known that growth-emphasized genotypes had a higher protein requirement and need a closer energy–protein ratio to exploit their growth potential (Emmans and Kyriazakis, 1997; Quiniou et al., 1996; Whittemore et al., 2001). Metabolic differences in protein disposition and energy metabolisms between the sire lines could be an explanation for the findings of the current study. Saintilan et al. (2015) reported 13 % higher average digestible lysine requirements for the 25 % most efficient pigs compared to the 25 % least efficient pigs. Furthermore, the effect of sire line might be attributable to differences in additive genetic variance and thus heritability between lines, leading to different breeding progress. It can be assumed that the genetic progress realized under the diet used on the test farm is not completely transferable when using other diets, like in the current trial. In this context, Godinho et al. (2018b, c) reported impairment of lipid disposition and residual energy intake in pigs on different diets due to genotype by feed interactions, which could be a reason for the observed differences in growth performance and carcass value. Moreover, findings of Škorput and Luković (2018) suggest a range of additive genetic variance for ADG from 586.09±55.73 g d${}^{-1}$ in the Large White population to 1190.03±139 g d${}^{-1}$ in Piétrain populations.

As expected, heavier HCW was detected for sire line A due to faster growth rate. This is in line with Miar et al. (2014), who reported a strong correlation between growth rate and HCW (r=0.75±0.28; p≤0.05). According to Dufrasne et al. (2013) and Zumbach et al. (2007), with 2.03 % and 6.7 %, respectively, sire line explained only low proportions of genetic variance for final live body weight. It is even known that heritability ranges between 0.15 and 0.39 depending on the estimation method and the population (Dufrasne et al., 2014; Fragomeni et al., 2016; Holl et al., 2008; Khanal et al., 2019; Miar et al., 2014). Against expectations, the FT was equal in both sire lines. However, the ratio of LD and FT in consideration of sire line suggested a lower FT, which is resulting from a higher lean growth potential in pigs sired by line B. Additionally, the current results are in line with van Wijk et al. (2005), who reported that selection for higher growth led to effects decreasing most primal and sub-primal cut weights. Selection for leanness in Piétrain sire has resulted in reduced feed intake capacity (Labroue et al., 1999; Lean et al., 1972). Hence, the leaner carcass with lighter BE is linked to a higher muscle growth potential of pigs sired by sire line B. Findings of Correa et al. (2008) and Lowell et al. (2019) support these results. However, as discussed above, a higher protein requirement in combination with an inappropriate energy level could also be an explanation for the poorer carcass value of the offspring sired by line A. Furthermore, based on a nonlinear association between daily growth and muscle growth, pigs with more than 60 % lean meat tend to grow slower (Argemí-Armengol et al., 2019), which is consistent with the results of the current study.

**Table 6:** LSQ mean (with SE as index) of growth and carcass traits according to the birth weight classes (BW${}_{\text{Q}}$).

	BW${}_{\text{Q}}$		
	Q${}_{1}$	Q${}_{2}$	Q${}_{3}$
Growth trait			
ADG${}_{\text{net}}$ (g d${}^{-1}$)	499.47${}_{2.54}^{\text{a}}$	527.40${}_{2.39}^{\text{b}}$	541.76${}_{2.54}^{\text{c}}$
Carcass traits			
HCW${}^{1}$ (kg)	87.23${}_{0.25}^{\text{a}}$	91.89${}_{0.17}^{\text{b}}$	93.05${}_{0.25}^{\text{c}}$
LM${}^{2}$ (%)	59.00${}_{0.09}^{\text{a}}$	60.26${}_{0.06}^{\text{b}}$	60.97${}_{0.09}^{\text{c}}$
LD${}^{2}$ (mm)	63.70${}_{0.13}^{\text{a}}$	64.89${}_{0.09}^{\text{b}}$	65.49${}_{0.13}^{\text{c}}$
FT${}^{2}$ (mm)	14.78${}_{0.07}^{\text{a}}$	13.82${}_{0.05}^{\text{b}}$	13.29${}_{0.07}^{\text{c}}$
BE${}^{2}$ (kg)	13.26${}_{0.02}^{\text{a}}$	12.96${}_{0.01}^{\text{b}}$	12.79${}_{0.02}^{\text{c}}$
BLM${}^{2}$ (%)	56.31${}_{0.12}^{\text{a}}$	57.93${}_{0.08}^{\text{b}}$	58.81${}_{0.12}^{\text{c}}$
BH${}^{2}$ (kg)	17.33${}_{0.03}^{\text{a}}$	17.75${}_{0.02}^{\text{b}}$	17.95${}_{0.03}^{\text{c}}$
LO${}^{2}$ (kg)	10.88${}_{0.01}^{\text{a}}$	11.07${}_{0.009}^{\text{b}}$	11.17${}_{0.01}^{\text{c}}$
BLO${}^{2}$ (kg)	6.81${}_{0.01}^{\text{a}}$	7.00${}_{0.009}^{\text{b}}$	7.10${}_{0.01}^{\text{c}}$
BSH${}^{2}$ (kg)	8.52${}_{0.01}^{\text{a}}$	8.66${}_{0.007}^{\text{b}}$	8.73${}_{0.01}^{\text{c}}$

${}^{1}$ Slaughter age adjusted to 173 d. ${}^{2}$ HCW adjusted to 91 kg. ${}^{\text{a–c}}$ Values within a row with different superscripts differ significantly (p<0.001).

Males reached a higher BW${}_{\mathrm{final}}$ at the same slaughtering age as compared to females, which is linked to 35.5 g d${}^{-1}$ faster growth rate. Additionally, FCR was better by 0.11 in females, which agrees with the findings of Serrano et al. (2008) and Renaudeau et al. (2006), but is in contrast to Lee et al. (2019) and Peinado et al. (2008). The poorer FCR of males is related to the moderately faster growth rate and the 160 g d${}^{-1}$ higher ADFI. The PD${}_{\text{max}}$ is the point of maximum protein accretion of a pig and up to this plateau, protein growth responds linearly to feed intake (Campbell et al., 1985; Whittemore and Fawcett, 1976). Consequently, pigs with a high feed intake reach this point earlier and tend to accumulate fat tissue as the PD${}_{\text{max}}$ is exceeded (Lewis and Lee Southern, 2000; Tullis, 1982). This might explain the fatter carcasses of male pigs in the current study. Generally, it is expected that females have a higher ratio of protein accretion relative to lipid accretion, and this is in agreement with Noblet et al. (1999), Schinckel et al. (2008) and Serrano et al. (2008). Further insight into biological differences between the sire lines can be drawn from the significant interactions between sire line and sex, which were also found by Lowell et al. (2019) and Morales et al. (2011). Generally, these interactions clearly indicate that pigs sired by line A grew faster than pigs sired by line B and that pigs from sire line B showed a leaner carcass with heavier primal cuts compared to the other sire line. Similar growth rate (ADG${}_{\text{net}}$, ADG) of males and females sired by line A suggested a more homogenous growth performance in offspring of this sire line. However, the differences in carcass traits between males and females were smaller in sire line A and supported the homogenous growth of this line. Moreover, only females of sire line B differed in growth rate (ADG${}_{\text{net}}$, ADG) compared to all other variations, which is explained by the significant lower ADFI in comparison with males regardless of the sire line. Neither a difference in growth rate (ADG${}_{\text{net}}$, ADG) nor in ADFI between both male groups was detected, but females differed significantly. Regarding a different protein requirement between the sire lines, an explanation could be that the females sired by line A better exploited their growth potential compared to the males of the same sire line, despite the fact that the energy–protein ratio does not seem to be optimal for this genotype. Findings by Chen et al. (1999) and Hansen and Lewis (1993) indicated different responses with deficient or excessive protein intake between castrated males and females, and Martins et al. (2012) reported differences in protein requirements between different genotypes. Hence, a better adaptability to the available level of protein and energy content of females sired by line A in contrast to the males of the same genotype is an explanation for the interactions between sire line and sex regarding the growth rate.

Regarding the birth weight, the ADG${}_{\text{net}}$ increased with increasing BW${}_{\text{Q}}$ and indicated that heavier birth weight pigs grow faster with improving subsequent performance, which is in line with Fix et al. (2010) and Vázquez-Gómez et al. (2020). However, Douglas et al. (2013) reported that birth weight had less impact on subsequent growth performance compared to weaning weight or initial weight in the finisher barn. Nevertheless, in the current study fatter carcasses in pigs of the BW${}_{\text{Q1}}$ and BW${}_{\text{Q2}}$ compared to heavier birth weight pigs of BW${}_{\text{Q3}}$ were detected and are in agreement with the findings of Nissen et al. (2004) and Rehfeldt and Kuhn (2006). A decreasing proportion of ham, loin and belly in the lightest birth weight classes suggested disadvantages in growth performance, in terms of less ADG, lower ADFI and a poorer FCR. These findings were generally in line with Gondret et al. (2005) and Rehfeldt et al. (2008). However, lower belly weight in pigs with higher birth weight may be explained by further selection for higher proportion of lean meat. In agreement with Kim et al. (2017), stronger selection for lean meat is linked to less fat disposition in belly muscle and leads to lower belly weight. Moreover, no interactions between sire line and BW${}_{\text{Q}}$ indicated that offspring of both sire lines show the same biological relation between birth weight and carcass traits, except for LD. Hence, similar LD between the BW${}_{\text{Q2}}$ and the BW${}_{\text{Q3}}$ class in sire line A (p≥0.05) indicated that an additional gain of ≥1.70 kg birth weight would not lead to a further meat gain within this genetic line. Consequently, biological limit in this trait seems to be reached, and further selection in sire line A on birth weight would not increase the LD. According to Beaulieu et al. (2010) and Gondret et al. (2005), it is not unexpected that carcass leanness is not primarily influenced by birth weight. Other factors, such as initial and final body weight, the level of feed intake, growth rate, feed processing and delivery, and genetic potential were also involved (Patience et al., 2015). This interaction supported a difference in protein and energy metabolism between the sire lines.

Based on the findings of Latorre et al. (2003), Lloveras et al. (2008), Lowell et al.  (2019) and Miller et al. (2000), pH values in this study were generally within normal range. Unlike Miller et al. (2000) and Latorre et al. (2003) but in agreement with Kim et al. (2017) and Fecke (2013), pH${}_{\text{45 min}}$ was not affected by genotype. However, pigs sired by sire line B showed lower pH${}_{\text{24 h}}$, indicating poorer meat quality. Drip losses are moderately to strongly negatively correlated with the texture of loin (-0.23±0.06), belly weight (-0.46±0.14) and pH${}_{\text{24 h}}$ (-0.99±0.49) (p≤0.05) (Miar et al., 2014). Similar results were reported by Kim et al. (2017) and Otto et al. (2006). Consequently, poorer water retention capacity among offspring of sire line B seems likely, and the leaner carcasses of this offspring back up this result. It suggests that higher leanness in line B and in females means poorer meat quality.

## 5 Conclusions

This research confirms differences in growth performance and carcass characteristics between crossbreeds of two different sire lines. Pigs sired by line A grow faster and had higher ADFI and the same FCR compared to offspring of line B. This resulted in a fatter carcass and heavier belly weights, which indicated a better meat quality due to a higher pH${}_{\text{24 h}}$ in crossbreeds sired by line A. Leaner carcasses with heavier primal cuts were detected at offspring of line B and at heavier birth weight pigs. Males grew faster and reached a heavier but also fatter carcass. Regardless of sire line, piglets with heavier birth weight were leaner and had heavier primal and boneless primal weights. Except for LD, no interactions between sire line and BW${}_{\text{Q}}$ were detected, indicating subsequent growth was equal in both lines, depending on birth weight. The detected interaction for LD between sire line and BW${}_{\text{Q}}$ as well as sire line by sex interactions indicated different protein requirements between the genotypes. In conclusion, in a fatting production system, paternal line A is preferable if a producer wishes to increase the number of fatting pigs per fattening place and year. Additionally, in the dam line, emphasis should be put on leanness. Breeding with paternal line B is a good option for a finisher producer if the goal is to increase leanness and carcass primal cuts.

## Data Availability

All necessary data are provided within the article.
